# Quantitative Prediction and Kinetic Modelling for the Thermal Inactivation of *Brochothrix thermosphacta* in Beef Using Hyperspectral Imaging

**DOI:** 10.3390/foods14162778

**Published:** 2025-08-10

**Authors:** Qinglin Li, Juan Francisco García-Martín, Fangchen Ding, Kang Tu, Weijie Lan, Changbo Tang, Xiaohua Liu, Leiqing Pan

**Affiliations:** 1 College of Food Science and Technology, Nanjing Agricultural University, Nanjing 211800, China; 2023808107@stu.njau.edu.cn (Q.L.); kangtu@njau.edu.cn (K.T.); weijie.lan@njau.edu.cn (W.L.); tangcb@njau.edu.cn (C.T.); 2 Departamento de Ingenieria Química, Facultad de Quimica, Universidad de Sevilla, 41012 Sevilla, Spain; jfgarmar@us.es (J.F.G.-M.); fandin@alum.us.es (F.D.); 3 Sanya Institute of Nanjing Agricultural University, Sanya 572024, China; 4 Department of Biological and Food Engineering, Bozhou University, BoZhou 236800, China

**Keywords:** beef, *Brochothrix thermosphacta*, hyperspectral imaging, quantitative prediction, thermal inactivation kinetics

## Abstract

In this work, the feasibility of simulating the thermal inactivation of *Brochothrix thermosphacta* in beef during heating processing based on hyperspectral imaging (HSI) in the wavelength range of 400–1000 nm was investigated. The Weibull and modified Gompertz kinetic models for the thermal inactivation of *B. thermosphacta* in beef heated in the range 40–60 °C were developed based on the full wavelength, featured spectral variables, and their principal component scores of HSI information, respectively. Notably, the specific wavebands at 412 nm and 735 nm showed a strong correlation with the surviving *B. thermosphacta* population during the beef heating process. The partial least squares regression models had a satisfactory ability in quantifying *B. thermosphacta* in beef, with an R_v_^2^ and RMSE of 0.826 and 0.341 log CFU/g, respectively. Furthermore, the Weibull model coupled with the HSI at 735 nm was suitable for kinetic modeling of the thermal inactivation of *B. thermosphacta* in beef, with an R^2^ value of 0.937. Consequently, this work suggests the potential of the HSI technique for quantifying and monitoring microbes in meat during heating and can be applied for the thermal inactivation kinetic modeling of microorganisms.

## 1. Introduction

Microbial contamination, including pathogenic and spoilage bacteria, is one of the most important factors of foodborne diseases caused by eating meat products [1,2]. Beef is a nutrient-rich ideal medium for the growth of different microbes like *Brochothrix thermosphacta, Pseudomonas* spp., and *Enterobacteriaceae* [3]. *B. thermosphacta* is known as a common dominant spoilage bacterium in beef due to its putrefactive capability of producing volatile organic compounds, causing cheesy or sour odors [4,5]. Its growth and reproduction not only spoil meat but also pose a potential risk to human health by producing biogenic amines, especially in cases of significant contamination [6,7]. It is essential for beef quality and potential health threats to monitor *B. thermosphacta* loads in beef products during processing. Heating treatment is widely used for meat processing [8] and aims to strengthen sensory properties and destroy microorganisms in order to enable meat safety and extend meat shelf-life [9,10]. Thermal treatment has some negative effects on meat products, such as the loss of nutrients [11], poor quality attributes [12], and excessive energy consumption, posing the challenge of fully inactivating microorganisms while retaining original sensory quality and nutrients.

To address the above challenge, many researchers have focused on the specific thermal time and temperature obtained by constructing predictive survival models of bacteria. Weibull and Gompertz kinetic models are widely used in microbial prediction to describe growth processes over time. These two approaches are complementary and can be utilized to model complex dynamic systems. Thus, the thermal inactivation models of *Listeria monocytogenes* in crab meat were explored [13], and a Z-value of 4.9 °C was reported. Furthermore, the thermal inactivation of Shiga toxin-producing *Escherichia coli* in ground beef with varying fat content was researched [14], and the ranges of the obtained D-values were 15.93–11.69, 1.15–1.12, and 0.14–0.09 min at 55, 60, 65, and 68 °C, respectively. It was observed that the previous research usually used the traditional plate count method to build thermal inactivation models for monitoring the surviving bacteria in meat during heating. Given the rapid technological progress of machine-led and intelligent food production lines, the time-consuming, laborious, and destructive culture-based method was obsolete [15]. The latest nondestructive technologies must be utilized for bacterial detection to satisfy the development demand of the meat industry.

Hyperspectral imaging (HSI) is a promising technology for rapid nondestructive determination, which can simultaneously acquire both the spatial and spectral information of samples [16]. HSI can identify the spectral features related to the fundamental vibrations of molecular overtones and combinations; thus, it is widely applied for the quantitative and qualitative analysis of quality parameters (e.g., color, moisture content, and microbial loads) during food processing [17], for example, the quantified color (R_p_^2^ = 0.890) and moisture content (R_p_^2^ = 0.869) of beef during microwave heating based on HSI [18], and HSI was used to construct PLSR quantitative models of *Salmonella Typhimurium* (R_p_^2^ = 0.9687) and *Escherichia coli* (R_p_^2^ = 0.9687) in pork ultrasonicated for 10, 20, and 30 min [19]. Although the application of the HSI technology in the thermal processing of meat was emphasized in several recent studies [20,21], few studies have combined HSI with thermal inactivation models to explore the thermal inactivation state of microorganisms in meat during heating in order to precisely determine the thermal parameters for achieving commercially sterile meat products.

Therefore, to explore the feasibility of simulating the thermal inactivation of *B. thermosphacta* in beef during heating based on HSI, the aims of this study are to (i) investigate the hyperspectral spectral characteristic in the 400–1000 nm spectral range of beef inoculated with *B. thermosphacta* during heating at 40, 45, 50, 55, and 60 °C; (ii) construct thermal inactivation models for the quantitative prediction of *B. thermosphacta* in beef based on HSI; and (iii) perform kinetic modeling for the thermal inactivation of *B. thermosphacta* in beef heated in the range 40–60 °C based on the feature HSI information extracted by three methods, and perform a comparison with those models based on the conventional plate count method.

## 2. Materials and Methods

### 2.1. Inoculum Preparation

*B. thermosphacta* (strain number: ACCC03872) was stored at 4 °C in the laboratory refrigerator until use (College of Food Science and Technology, Nanjing Agricultural University, Nanjing, Jiangsu, China). The bacterial cultures were obtained by incubating *B. thermosphacta* on nutrient agar at 30 °C for 24 h, followed by subculturing under the same conditions. For the sake of bacterial suspensions, the cultures were washed and suspended with a sterile saline solution (0.85% NaCl *w*/*v*) to a cell level of approximately 10^7^–10^8^ CFU/mL (OD_600_ = 0.39). The well-prepared bacterial suspensions were applied as inoculum to the beef samples.

### 2.2. Sample Preparation and Inoculation

Beef samples (*Longissimus dorsi*) purchased from the local supermarket were transported to the laboratory in an average time of 30 min. Beef was trimmed to remove the fat or connective tissue. After wiping with ethanol (75% *v*/*v*), beef samples were treated with ultraviolet light (UV-C) for 30 min. Then, samples were aseptically divided into 4 cm × 4 cm × 2 cm (length × width × thickness) pieces using a sterile knife, thoroughly mixed with the inoculum for 3 min, and naturally dried on the sterile table for 30 min to make bacteria adhere to meat surfaces. The inoculated samples were heat-sealed in polyethylene plastic bags (0.08 mm in thickness).

### 2.3. Thermal Inactivation

For the packaged samples, the submerged heating treatments at different temperatures were carried out in a constant temperature water bath (HH-8, Changzhou Guohua Electric Appliance Co., Ltd., Changzhou, China). At each time interval, 10 samples were taken for the heating treatment, particularly, at 24 min intervals at 40 °C for 120 min, at 12 min intervals at 45 °C for 60 min, at 6 min intervals at 50 °C for 30 min, at 3 min intervals at 55 °C for 15 min, and at 0.5 min intervals at 60 °C for 2.5 min. Thus, 60 samples were obtained for each temperature treatment; a total of 300 samples were used for this experiment. The come-up time based on the pre-experiments was excluded from the total heating time. At each time interval, 10 samples were taken for the heating treatment. Immediately, the heated samples were plunged into the ice bath to prevent further inactivation. The HSI data collection and surviving *B. thermosphacta* enumeration for beef samples were subsequently performed. Finally, a total of 300 samples were used for this experiment.

### 2.4. Enumeration of Surviving B. thermosphacta

The conventional plate count method was applied for the enumeration of surviving *B. thermosphacta* in heated samples. About 5 g of each beef sample was transferred to a sterile homogenization bag with 45 mL of 0.85% NaCl autoclaved solution and homogenized for 2 min. Serial 10-fold dilutions were made, and 0.1 mL were plated on streptomycin thallous acetate actidione agar (STAA) containing the STAA selective supplement. Then, the STAA plates were incubated at 30 °C for 48 h. The enumeration of surviving *B. thermosphacta* was described as ten-based logarithm values (log CFU/g).

### 2.5. Modeling B. thermosphacta Inactivation in Beef

#### 2.5.1. Mathematical Models of *B. thermosphacta* Inactivation

The inactivation curves of *B. thermosphacta* were described using primary models, including the Weibull model [22] and modified Gompertz model [23]. Secondary models were used to describe the effect of temperature on the inactivation parameters. For the Weibull model (Equation (1)), a suitable secondary model for the parameter δ was expressed as Equation (2). The parameters $k_{m}$ and L obtained from the modified Gompertz model (Equation (3)) were fitted with Equations (4) and (5), respectively.

(1) $${log}_{10}\frac{N}{N_{0}}=-{\left(\frac{t}{\delta }\right)}^{\eta }\;$$

(2) $$\log_{10}\delta =a\times T+c\;$$

In which $N_{0}$ and N are the bacterial population or hyperspectral characteristic values (log CFU/g or none) at time 0 and actual time *t* (min), respectively; δ is the time parameter or time of first decimal reduction; η is the shape parameter; T is the temperature (°C); and a and c are model parameters.

(3) $$\log_{10}\frac{N}{N_{0}}=-A\;\times \;exp\left\{-exp\left[\frac{k_{max}\times \exp\left(1\right)}{A}\times \left(L-t\right)+1\right]\right\}$$

(4) $$k_{m}={\left[C_{1}\times (T-T_{min})\right]}^{2}$$

(5) $$L={\left[C_{2}\times (T-C_{3})\right]}^{2}$$

where $N_{0}$, N, T, and t are the same as expressed above; A is the asymptotic survival ratio reached at the end; $k_{max}$ is the maximum inactivation rate (time^−1^); L is the shoulder length (min); T_*min*_ is the theoretical minimum temperature for inactivation (°C); and $C_{1}$, $C_{2}$, and $C_{3}$ are model parameters.

#### 2.5.2. One-Step Nonlinear Regression

Primary and secondary models were constructed simultaneously by a one-step kinetic analysis. All the inactivation data were assembled and analyzed simultaneously using the nonlinear regression procedure (FITNLM) in MATLAB R2016a software (The Mathworks, Inc., Natick, MA, USA). This approach, utilizing advanced computational tools for simultaneous parameter estimation, is integral to the continuing research aimed at enhancing our understanding and predictive capabilities regarding microbial inactivation in food safety, particularly through the integration of hyperspectral imaging with kinetic modeling. Based on nonlinear least squares optimization algorithms, the FITNLM procedure was designed to estimate inactivation parameters in primary and secondary models, simultaneously. Inactivation parameters of the Weibull and modified Gompertz models were expressed as Equation (6) and Equation (7), respectively.

(6) $$\{P\}\;={\left\{a,\;c,\;\eta \right\}}^{\prime }$$

(7) $$\{P\}\;={\left\{A,\;C_{1},\;C_{2},\;C_{3},\;T_{\min}\right\}}^{\prime }$$

### 2.6. Hyperspectral Imaging (HSI) Data Acquisition and Preprocessing

Prior to *B. thermosphacta* enumeration, a hyperspectral reflectance imaging system (SPECIM, Oulu, Finland) in the range of 400–1000 nm was used to acquire the HSI data of beef samples. The system was composed of a CCD camera with a spectral resolution of 2.8 nm, a 150 W halogen lamp, a sample delivery platform, and a computer with data acquisition software. The entire hyperspectral imaging system was enclosed in a black box to eliminate the influence of external light during data acquisition.

The working parameters of the HSI system were as follows: The lamp was fixed at around a 45° angle over the sample and positioned at a distance of about 30 cm from the sample; the camera exposure time was set as 3 ms; and the speed of the sample delivery platform was 7.23 mm/s. The size of collected hyperspectral images of beef samples was 804 × 440 pixels, retaining the spectral information of 420 available bands in the range of 400–1000 nm.

The camera dark currents and external factors have an impact on the collected images, so it is necessary to calibrate raw hyperspectral images. Calibrated images were acquired by a white reference image collected from a Teflon whiteboard (99% reflectivity) and a dark reference image collected by covering the camera lens with its black opaque cap. The calibrated image was calculated by Equation (8):

(8) $$I_{cal}\;=\;\frac{I_{raw}-I_{dark}}{I_{white}-I_{dark}}$$

where $I_{cal}$ is the calibrated image; $I_{raw}$ is the raw image; $I_{white}$ is the white reference image; and $I_{dark}$ is the dark reference image.

Spectral extraction was conducted in the MATLAB R2016a software. The region of interest (ROI) based on the whole sample surface was isolated from the background by the threshold segmentation algorithm. The mean spectral values of all pixels in each ROI were considered as spectral information of each beef sample.

To minimize the noise interference from the instrument itself and the surroundings, the extracted spectra were preprocessed. Several algorithms, including orthogonal signal correction (OSC), standard normal variate (SNV), and multiplicative scattering correction (MSC), were compared.

### 2.7. Modeling of Spectral Information from HSI

#### 2.7.1. Quantitative Modeling of Surviving *B. thermosphacta*

Partial least squares regression (PLSR) and support vector machine regression (SVMR) were used for the quantitative prediction of surviving *B. thermosphacta*. The PLSR model, as a multilinear regression model, can identify linear relationship between observations and predictions [24]. SVMR is a supervised learning method using the concept of decision planes with an excellent generalization ability, which can build nonlinear relationships between lossless signal rationalization indicators [25]. Both are widely applied in the regression of spectral data.

In the work, 300 spectral data were randomly divided into a calibration dataset and validation dataset at a ratio of 3:1 using the Kennard–Stone algorithm in MATLAB R2016a software. Then, PLSR and SVMR prediction models were established based on the preprocessed full-band spectral data. The predicted performance of different quantitative models was compared.

#### 2.7.2. Survival Kinetic Modeling of *B. thermosphacta* by HSI

**Figure 1:**
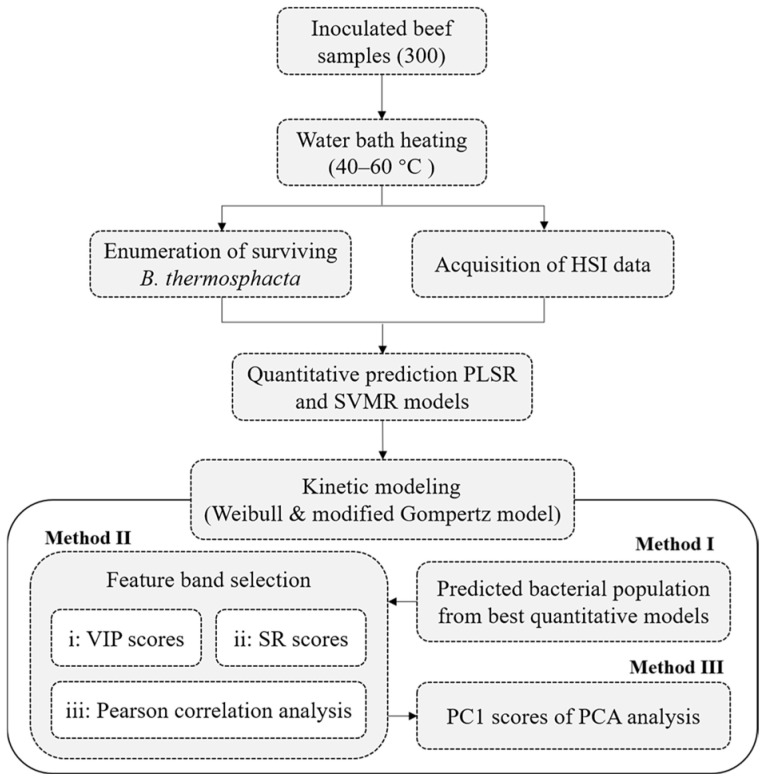
Flowchart of quantitative prediction and kinetic modeling for the thermal inactivation of *B. thermosphacta* in beef using HSI.

In this work, three methods were adopted to build survival kinetic models of *B. thermosphacta* based on HSI information (Figure 1):

Method I: The surviving bacterial population predicted by the above optimal quantitative models (PLSR or SVMR model) was treated as input values to the above mathematical models describing the thermal inactivation of *B. thermosphacta*.

Method II: The feature spectra reflecting the microbial population were used for the construction of the survival model of *B. thermosphacta*. Hyperspectral data inevitably contains plenty of redundant and useless information, which affects information extraction, adds the burden of data processing, and easily makes models less effective and stable. Therefore, it is necessary to select feature bands such as variable importance in the projection (VIP), selectivity ratio (SR), Pearson correlation analysis, and continuous projection algorithm. Among them, both VIP and SR scores depend on the PLSR model to measure the importance of wavelength variables to the predicted variables. Generally, the VIP score is taken as 1.0 and recognized as feature bands when the VIP is higher than 1.0. And a higher SR score means that the band is more important, and the contribution is the greatest to the model [26]. In addition, the correlation between the whole wavelengths and bacterial populations could be judged by a Pearson correlation analysis. The spectral values at the band with the largest correlation coefficient have the strongest correlation with the bacterial concentration [27]. Method II would use VIP scores, SR scores, and a Pearson correlation analysis to screen the feature bands, and the spectral values at the selected characteristic bands would be substituted into the above mathematical models for survival kinetic modeling.

Method III: The survival kinetic model of *B. thermosphacta* was constructed based on the first principal component (PC1) of the full-band HSI data. A principal component analysis (PCA) could also compress hyperspectral data to reduce data redundancy and improve the accuracy and stability of models [28]. PC1 could be obtained by the PCA, which could interpret the most spectral information. Therefore, Method III used PC1 as the input values of the survival function of *B. thermosphacta* to construct its survival kinetic model.

### 2.8. Evaluation of Survival Models

It is essential to evaluate the performance of quantitative models, which directly determine the accuracy of survival kinetic models. Generally, the performance of quantitative prediction models is evaluated by the coefficients of determination in calibration (R_c_^2^) and validation (R_v_^2^), the corresponding root mean square error (RMSEC and RMSEV), and the ratio of performance to deviation (RPD) [29].

The goodness of fit of survival kinetic models was assessed by the coefficient of determination (R^2^) and root mean square error (RMSE) [30]. In addition, the Akaike Information Criteria (AIC) can also help to judge the goodness of fit for the model. A model with a low AIC can perform better in prediction.

### 2.9. External Validation of Survival Kinetic Models

The survival kinetic models were validated by two extra experiments at 40 °C for 120 min (30 samples) and 60 °C for 2.5 min (30 samples). In order to compare the difference between the observations and predictions of survival kinetic models based on validation experiments at 40 °C and 60 °C, the accuracy ($A_{f}$ Equation (9)) and bias ($B_{f}$, Equation (10)) factors proposed elsewhere [31] were taken into account. When both $A_{f}$ and $B_{f}$ are close to one, the growth model is highly reliable.

(9) $$A_{f}=10^{\frac{\sum_{i=1}^{n}\left|log(x_{pre}{/x}_{obs})\right|}{n}}\;$$

(10) $$B_{f}={\;10}^{\frac{\sum_{i=1}^{n}log(x_{pre}{/x}_{obs})}{n}}$$

In Equations (9) and (10), $x_{pre}$ and $x_{obs}$ represent the predicted and experimental values, respectively; n is the total number of the experimental data points.

## 3. Results and Discussion

### 3.1. Analysis of Spectral Features

**Figure 2:**
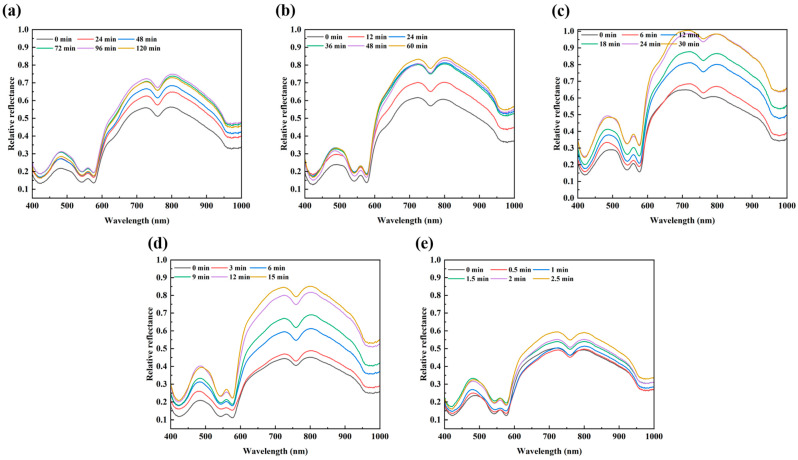
Mean spectra of beef samples at different heating times under different temperatures ((**a**): 40 °C; (**b**): 45 °C; (**c**): 50 °C; (**d**): 55 °C; and (**e**): 60 °C).

Figure 2 shows the mean reflectance spectra of beef samples at different heating times under different temperatures. The general trends of spectra at different temperatures were similar and the spectral values increased gradually with increased heating time, which was similar to the trends of reflectance spectra of beef slices pretreated with drying at different temperatures [32]. This trend may be associated with factors such as protein, water loss, and color change.

As seen in Figure 2, several characteristic peaks and valleys can be highlighted in the 400–1000 nm range. An evident valley around 420 nm was related to the Soret absorption, resulting from the porphyrin compounds [33]. The bands at approximately 490, 545, and 580 nm were associated with metmyoglobin [34], oxymyoglobin [35], and respiratory pigments (e.g., myoglobin, deoxymyoglobin, and hemoglobin) [36], respectively. Two absorption bands at 760 and 970 nm were, respectively, correlated with the third and second overtone O–H stretching, corresponding to the water absorption [37,38]. The presence of characteristic peaks in mean spectra indicated the possibility of using HSI information to determine the variations in protein and water content of beef during heating. Liu et al. [18] have investigated HSI (400–1000 nm) to correlate the mean spectra of beef samples and moisture content and color (L*, a*, and b*) during microwave treatment and demonstrated the ability of HSI for monitoring the changes in some quality parameters during microwave heating. It is known that the reduction in microbial counts of beef samples during heat treatment accompanied changes in quality parameters such as moisture and protein. Thus, bacterial populations might be indirectly quantified by clarifying the correlations between bacterial populations and the protein or water content of beef samples during heating.

### 3.2. Quantitative Prediction of Surviving B. thermosphacta by HSI

The mean reflectance spectra were used to construct quantitative prediction models of surviving *B. thermosphacta* by PLSR and SVMR (Table 1). In terms of untreated full-band spectra in the 400–1000 nm range, the overall prediction results were roughly acceptable. The R_v_^2^ of PLSR and SVMR were, respectively, 0.780 and 0.775, and the corresponding RMSEV were, respectively, 0.341 log CFU/g and 0.354 log CFU/g. After preprocessing, the predicted accuracy of both PLSR and SVMR models was slightly but insignificantly improved. As for PLSR models, the MSC algorithm provided the best modeling performance with R_v_^2^ = 0.826, RMSEV = 0.341 log CFU/g, and RPD = 2.415. The SNV algorithm in SVMR models performed best with an R_v_^2^ of 0.804, RMSEV of 0.354 log CFU/g, and RPD of 2.275. Apparently, the MSC-PLSR model presented the optimal capability for predicting surviving *B. thermosphacta.*

### 3.3. Survival Kinetic Modeling of B. thermosphacta by the Plate Count Method

**Figure 3:**
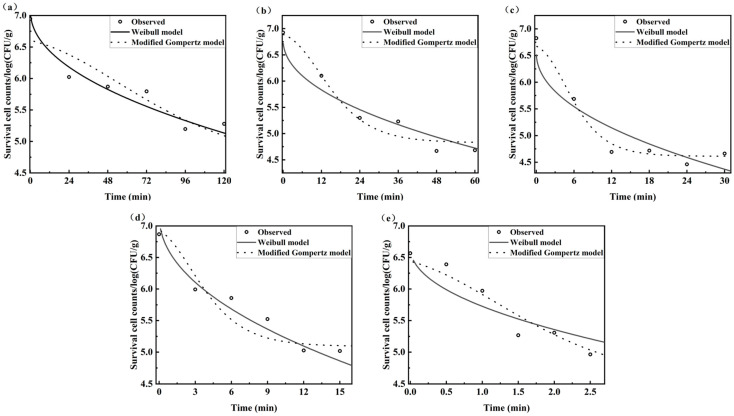
Heat survival curves of *B. thermosphacta* at different temperatures based on the plate count method ((**a**): 40 °C; (**b**): 45 °C; (**c**): 50 °C; (**d**): 55 °C; and (**e**): 60 °C).

The traditional plate count method, a gold standard method for bacterial detection, was used as a reference method to construct Weibull and Modified Gompertz models for the survival kinetics of *B. thermosphacta*. Figure 3 exhibits the heat survival curves of *B. thermosphacta* at different temperatures based on the plate count method. In the temperature range of 40–60 °C, the populations of *B. thermosphacta* initially declined sharply and then decreased very slowly with increased time, with the microbial reductions in two orders of the magnitude [39]. The deactivation degree of *B. thermosphacta* relied on temperature and the holding time at that temperature. Overall, the survival curve had an insignificant shoulder effect and displayed a nonlinear downward region followed by pronounced tailing. It indicated that the heat processing conditions for *B. thermosphacta* should be determined by the nonlinear rather than linear equations to avoid considerable errors. Nevertheless, existing research have not yet provided a satisfactory explanation for the phenomenon of the shoulder effect and tailing [40].

Table 2 records survival parameters fitted with Weibull and modified Gompertz models. All estimated parameters, other than C_2_, were statistically significant (*p* < 0.05). Like all inverse problems, the nonlinear regression was not able to ensure all parameters were accurate [41]. The shape parameter η of the Weibull models was independent on temperature and estimated as 0.749 (η < 1), indicating that all survival curves were concaved downward [42]. The great performance of the Weibull model was proven by an RMSE of 0.243 log CFU/g, R^2^ of 0.921, and AIC of −78.186. As for the modified Gompertz model, the minimum survival temperature (*T_min_*) was predicted as 36.395 °C. In addition, the RMSE, R^2^, and AIC corresponded to 0.242 log CFU/g, 0.929, and −78.434, respectively. Results suggested that both the Weibull model and modified Gompertz model were appropriate to describe the survival kinetics of heat treated *B. thermosphacta.*

### 3.4. Different Survival Kinetic Modeling Methods of B. thermosphacta

#### 3.4.1. Survival Kinetic Modeling of *B. thermosphacta* by Method I

The survival kinetic modeling was conducted based on the predictions of *B. thermosphacta* from the best MSC-PLSR model (Method I), as shown in Table 3. Similarly, parameters, except C_2_, fitted by the Weibull and modified Gompertz models differed significantly from zero (*p* < 0.05), and the η of 0.481 and *T_min_* of 36.009 °C were nearly identical to those by the traditional plate count method. The difference was that the prediction accuracy of the modified Gompertz model (R^2^ = 0.864, RMSE = 0.256 log CFU/g, and AIC = −73.919) was acceptable but lower than the Weibull model (R^2^ = 0.922, RMSE = 0.194 log CFU/g, and AIC = −91.699) by Method I and models by the plate count method. It indicated that the Weibull model was extremely accurate when applied for the description of the individual survival curves [43].

#### 3.4.2. Survival Kinetic Modeling of *B. thermosphacta* by Method II

**Figure 4:**
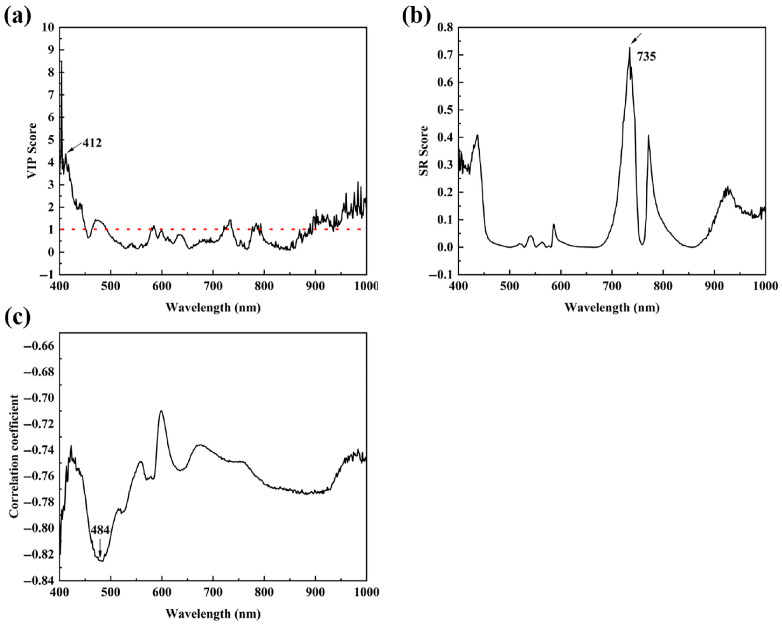
Results for the selection of feature wavelengths (**a**) variable importance in projection (VIP) scores and (**b**) selectivity ratio (SR) scores calculated by the PLSR; (**c**) Pearson correlation coefficients between *B. thermosphacta* population and spectral values.

The Weibull model by Method I was relatively accurate but had the obvious disadvantage of the need for the construction of PLSR models, which undoubtedly increased the computational effort and the risk of error accumulation. So, Method II directly fitted the thermal inactivation curves based on the feature bands reflecting the contents of *B. thermosphacta.* Figure 4 shows the VIP (Figure 4a) and SR scores (Figure 4b) from the MSC-PLSR model, and the Pearson correlation coefficients (Figure 4c) between the surviving *B. thermosphacta* population and HSI data. The bands at 412 nm and 735 nm, respectively, showed the largest VIP (>1) and SR scores, and the band at 484 nm had the strongest correlation with the surviving *B. thermosphacta* population. The results for thermal inactivation models built by the above three bands are listed in Table 4. Both Weibull and modified Gompertz models obtained using the 412 nm wavelength were unsatisfactory due to the relatively low predictive performance with the R^2^ of 0.753 and 0.676, respectively. The models obtained with the HIS data from 735 nm and 484 nm had a good predictive ability, with the R^2^ from 0.873 to 0.937, RMSE from 0.029 to 0.037, and AIC from −204.592 to −191.115. Among them, the model based on feature spectral values at 735 nm performed best, and its R^2^ and *T_min_* were, respectively, 0.937 and 36.471 °C, comparable to models by the plate count method. A similar situation to that of Method I was that the predictive accuracy of all Weibull models was higher than that of the modified Gompertz models at three different feature bands.

#### 3.4.3. Survival Kinetic Modeling of *B. thermosphacta* by Method III

Although the individual characteristic band had been screened in Method II for survival kinetic modeling of *B. thermosphacta*, the information contained in the single band was limited. The PCA for full-band spectra in 400–1000 nm was conducted to gain the PC1 fully representing the original spectral information, which allowed for a noticeable reduction in the dimensionality of the HSI data while retaining more information about the loads of microorganisms [44]. Survival kinetic models were developed based on the gained PC1, accounting for 92.3% of the spectral variability (Table 5). As for the model performance, the R^2^, RMSE, and AIC of the Weibull model were 0.918, 3.450, and 80.998, respectively, indicating a good predictive ability. And the corresponding evaluation results for the modified Gompertz model were 0.855, 4.700, and 99.549, with the *T_min_* of 36.527 °C close to that by the plate count method. Obviously, the Weibull model was still more accurate than the modified Gompertz model.

#### 3.4.4. Secondary Model of *B. thermosphacta*

**Figure 5:**
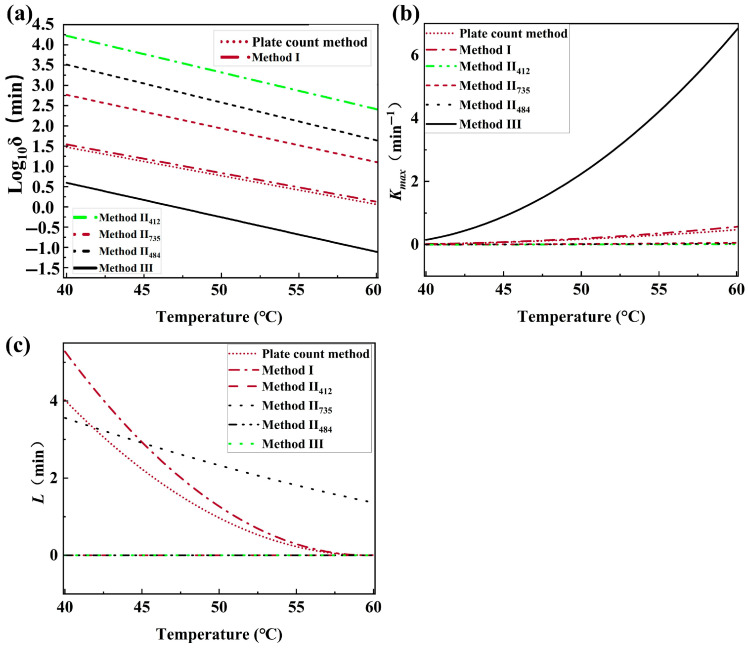
Effect of temperature on Weibull and modified Gompertz kinetics parameters of *B. thermosphacta*. (**a**) relationship between temperature and thermal inactivation parameter *δ*; (**b**) relationship between temperature and *K_max_*; (**c**) relationship between temperature and *L*.

Figure 5 depicts the effect of temperature on the Weibull and modified Gompertz kinetics parameters of *B. thermosphacta* inactivation. δ fitted with Weibull models was the scale parameter and log10 δ decreased linearly with increased temperature (Figure 4a). The maximum survival rate (K_min_, min^−1^) and the shoulder length (L, min) fitted with the modified Gompertz model showed, respectively, an upward and downward concave when the temperature increased (Figure 4b,c). It was observed that the trend of survival parameters (log10 δ, K_min_, and L) obtained by the traditional plate count method and HSI (Method I, II, and III) was similar as the temperature increased. However, the difference in magnitude made the curves of the survival parameters obtained from Method II and Method III as a function of temperature somewhat different.

A shorter time was needed for the thermal inactivation of bacteria at higher temperatures [43]. There was little available information to explain the dependance of L on temperature, which resulted in difficulties related to modeling the temperature effect. The demonstrated explanation included the growth stage of bacteria for the thermal survival research, the recovery media, and so on [45]. However, the relationship between δ, K_min_, L, and temperature could provide a theoretical reference for the thermal inactivation conditions of spoilage bacteria in meat.

#### 3.4.5. Validation of Survival Kinetic Models at Different Temperatures

The extra experimental data at 40 °C and 60 °C were employed to validate the survival kinetic models based on the plate count method and HSI, the results of which are listed in Table 6. It is important to highlight that the HSI values exhibit an inverse correlation with the viable counts following heating: as the viable counts decrease due to thermal inactivation, the HSI values increase. This relationship is critical for understanding the model outcomes. As for the Weibull and modified Gompertz models by the traditional plate count method, the ranges of $B_{f}$ and $A_{f}$ were 1.001–1.004 and 1.031–1.046, respectively, suggesting accurate and reliable survival models. The $B_{f}$ of models based on HSI data (Method I, II_735 nm_, and III) ranged from 0.999 to 1.004, 1.011 to 1.124, and 0.883 to 1.081, and the corresponding $A_{f}$ were 1.019–1.041, 1.053–1.129, and 1.174–1.341, respectively. If $A_{f}$ > 1.30, the model is ‘unacceptable’ [46], which meant that models built by PC1 scores (Method III) were unreliable and unacceptable. It seemed that Method I and II_735 nm_ were more suitable than Method III to build thermal inactivation models based on HSI information.

## 4. Conclusions

In this study, quantitative prediction and kinetic modeling for the thermal inactivation of *B. thermosphacta* in beef heated at 40–60 °C were performed using HSI information in the 400–1000 nm spectral range. The results indicated that spectral values of beef samples increased gradually when increasing heating time under different temperatures. The predictive MSC-PLSR model performed best in quantifying *B. thermosphacta* in beef during heating, with an R_v_^2^, RMSEV, and RPD of 0.826, 0.341 log CFU/g, and 2.415, respectively. In addition, Weibull models based on HSI information extracted by Method I and Method II_735 nm_ were suitable for kinetic modeling of the thermal inactivation of *B. thermosphacta* in beef, with R^2^ values of 0.907 and 0.937, which are comparable to those of models based on the traditional plate count method. It was suggested that HSI has the potential to quantify and monitor microbes in meat during heating and can be applied for the thermal inactivation kinetic modeling of microorganisms in meat, which provides the reference for the application of the HSI technique in predictive inactivation models. However, it is important to acknowledge the limitations of this method. While HSI shows promise, it is not yet a replacement for traditional viable plate count methods. Much more research by independent laboratories is necessary to validate the accuracy and reliability of HSI for microbial quantification and inactivation modeling. Instead, it should be employed in conjunction with generally accepted methods, such as plate counting, to ensure robust and accurate assessments of microbial inactivation.

## Figures and Tables

**Table 1:** PLSR and SVMR models of surviving *B. thermosphacta* in heated beef samples based on full-band spectra.

Modeling Methods	Pretreatment	Calibration		Internal Validation		RPD
		R_c_^2^	RMSEC	R_v_^2^	RMSEV	
			(log CFU/g)		(log CFU/g)	
PLSR	None	0.838	0.305	0.780	0.389	2.147
	OSC	0.838	0.304	0.784	0.388	2.167
	SNV	0.859	0.284	0.824	0.343	2.401
	MSC	0.859	0.284	0.826	0.341	2.415
SVMR	None	0.856	0.288	0.775	0.393	2.123
	OSC	0.828	0.315	0.784	0.366	2.167
	SNV	0.924	0.211	0.804	0.354	2.275
	MSC	0.857	0.288	0.770	0.383	2.100

Notes: The pretreatment included no preprocessing (None), orthogonal signal correction (OSC), standard normal variate (SNV), and multiplicative scattering correction (MSC). R_c_^2^: determination coefficient of the calibration dataset; RMSEC: root mean square error of the calibration dataset; R_v_^2^: determination coefficient of the internal validation set; and RMSEV: root mean square error of the internal validation test.

**Table 2:** Survival kinetic parameters of *B. thermosphacta* in beef estimated by Weibull and modified Gompertz models using the plate count method.

Model	Parameter	Estimate	Standard Error	*p* Value	RMSE	R^2^	AIC
Weibull	*a*	−0.071	0.008	$1.318\times 10^{-8}$	0.194	0.922	−91.699
	*c*	4.366	0.413	$4.391\times 10^{-10}$			
	η	0.481	0.064	$1.496\times 10^{-7}$			
Modified Gompertz	*A*	1.905	0.266	$6.068\times 10^{-7}$	0.256	0.864	−73.919
	$C_{1}$	−0.031	0.004	$4.860\times 10^{-7}$			
	$C_{2}$	−0.117	0.138	$4.069\times 10^{-1}$			
	$C_{3}$	59.601	13.114	$1.318\times 10^{-8}$			
	*T_min_* (°C)	36.009	0.870	$1.971\times 10^{-4}$			

Notes: *a*, *c*, *C*_1_, *C*_2,_ and *C*_3_ were model parameters. η: the shape parameter of Weibull model; *A*: the asymptotic survival ratio reached at the end; *T_min_*: the theoretical minimum temperature for inactivation; RMSE: the root mean square error; R^2^: the coefficient of determination; and AIC: Akaike Information Criteria to judge the goodness of fit for survival kinetic models.

**Table 3:** Estimated parameters of the thermal inactivation models for *B. thermosphacta* in beef based on predicted colony counts from HSI (Method I).

Model	Parameter	Estimate	Standard Error	*p* Value	RMSE	R^2^	AIC
Weibull	*a*	−0.074	0.194	$5.438\times 10^{-8}$	0.274	0.907	−70.982
	*c*	4.557	0.461	$1.503\times 10^{-9}$			
	η	0.560	0.899	$2.844\times 10^{-6}$			
Modified Gompertz	*A*	2.114	0.209	$2.664\times 10^{-9}$	0.258	0.926	−74.592
	$C_{1}$	−0.034	0.005	$1.093\times 10^{-6}$			
	$C_{2}$	−0.092	0.059	$1.371\times 10^{-1}$			
	$C_{3}$	66.786	8.451	$1.408\times 10^{-7}$			
	*T_min_* (°C)	36.308	0.781	$7.518\times 10^{-22}$			

Notes: *a*, *c*, *C*_1_, *C*_2_, and *C*_3_ were model parameters. η: the shape parameter of Weibull model; *A*: the asymptotic survival ratio reached at the end; *T_min_*: the theoretical minimum temperature for inactivation; RMSE: the root mean square error; R^2^: the coefficient of determination; and AIC: Akaike Information Criteria to judge the goodness of fit for survival kinetic models.

**Table 4:** Estimated parameters of the thermal inactivation models for *B. thermosphacta* in beef based on feature spectral values from HSI (Method II).

**Method**	Model	Parameter	Estimate	Standard Error	*p* Value	RMSE	R^2^	AIC
Method II_412_	Weibull	*a*	−0.906	0.015	$6.307\times 10^{-6}$	0.016	0.753	−241.415
		*c*	7.848	1.024	$1.196\times 10^{-7}$			
		η	0.606	0.159	$9.682\times 10^{-4}$			
	Modified Gompertz	*A*	0.223	0.513	$6.684\times 10^{-1}$	0.018	0.676	−233.207
		$C_{1}$	−0.005	0.001	$1.074\times 10^{-4}$			
		$C_{2}$	$-8.146\times 10^{-5}$	1236.900	1.000			
		$C_{3}$	56.260	0.019	$1.316\times 10^{-60}$			
		*T_min_* (°C)	37.924	2.079	$2.338\times 10^{-14}$			
Method II_735_	Weibull	*a*	−0.083	0.006	$5.024\times 10^{-13}$	0.037	0.937	−191.115
		*c*	6.085	0.309	$1.900\times 10^{-15}$			
		η	1.087	0.149	$2.755\times 10^{-7}$			
	Modified Gompertz	*A*	0.374	0.079	$1.351\times 10^{-4}$	0.041	0.924	−183.815
		$C_{1}$	−0.010	0.002	$4.480\times 10^{-6}$			
		$C_{2}$	−0.036	0.141	$8.013\times 10^{-1}$			
		$C_{3}$	92.435	131.640	$4.907\times 10^{-1}$			
		*T_min_* (°C)	36.471	0.916	$1.609\times 10^{-20}$			
Method II_484_	Weibull	*a*	−0.094	0.010	$1.705\times 10^{-9}$	0.023	0.915	−219.640
		*c*	7.253	0.580	$1.792\times 10^{-11}$			
		η	0.729	0.126	$8.239\times 10^{-6}$			
	Modified Gompertz	*A*	0.189	0.001	$4.732\times 10^{-6}$	0.029	0.873	−204.592
		$C_{1}$	−0.007	0.001	$5.856\times 10^{-6}$			
		$C_{2}$	−6.412	1.693	1.000			
		$C_{3}$	59.914	0.005	$4.318\times 10^{-72}$			
		*T_min_* (°C)	36.972	1.159	$2.796\times 10^{-19}$			

Notes: *a*, *c*, *C*_1_, *C*_2_, and *C*_3_ were model parameters. η: the shape parameter of Weibull model; *A*: the asymptotic survival ratio reached at the end; *T_min_*: the theoretical minimum temperature for inactivation; RMSE: the root mean square error; R^2^: the coefficient of determination; and AIC: Akaike Information Criteria to judge the goodness of fit for survival kinetic models.

**Table 5:** Estimated parameters of the thermal inactivation models for *B. thermosphacta* in beef based on PC1 scores of spectra from HSI (Method III).

Model	Parameter	Estimate	Standard Error	*p* Value	RMSE	R^2^	AIC
Weibull	*a*	−0.078	0.006	$3.835\times 10^{-12}$	3.450	0.918	80.998
	*c*	3.614	0.342	$4.412\times 10^{-10}$			
	η	0.862	0.115	$1.622\times 10^{-7}$			
Modified Gompertz	*A*	35.536	12.570	$1.010\times 10^{-2}$	4.700	0.855	99.549
	$C_{1}$	−0.078	0.015	$2.474\times 10^{-5}$			
	$C_{2}$	−0.0005	45.622	$9.999\times 10^{-1}$			
	$C_{3}$	63.927	0.001	$1.929\times 10^{-87}$			
	*T_min_* (°C)	33.846	1.562	$7.480\times 10^{-16}$			

Notes: *a*, *c*, *C*_1_, *C*_2_, and *C*_3_. η: the shape parameter of Weibull model; *A*: the asymptotic survival ratio reached at the end; *T_min_*: the theoretical minimum temperature for inactivation; RMSE: the root mean square error; R^2^: the coefficient of determination; and AIC: Akaike Information Criteria to judge the goodness of fit for survival kinetic models.

**Table 6:** The accuracy factor ($A_{f}$) and bias factor ($B_{f}$) from thermal inactivation models based on extra experimental data at 40 °C and 60 °C.

Model	Method	40 °C		60 °C	
		$B_{f}$	$A_{f}$	$B_{f}$	$A_{f}$
Weibull	Plate count method	1.001	1.031	1.004	1.046
	Method I	0.999	1.020	1.001	1.021
	Method II_735_	1.011	1.053	1.124	1.129
	Method III	1.014	1.341	0.898	1.295
Modified Gompertz	Plate count method	1.002	1.037	1.004	1.031
	Method Ⅰ	1.000	1.041	1.001	1.019
	Method Ⅱ_735_	1.014	1.060	1.022	1.062
	Method III	0.883	1.333	1.081	1.174

Notes: Method I, II_735,_ and III represented three different modeling methods. Methods I, II_735,_ and III were based on the predictions from the MSC-PLSR model, the spectral values at 735 nm, and the score values of the first principal component of the full spectra, respectively. The accuracy factor ($A_{f}$) and bias factor ($B_{f}$) referred to the difference between the observed values and predicted values from the extra experimental dataset at 40 °C and 60 °C.

## Data Availability

Data are contained within this article.
